# Hepatic arteriovenous malformation

**DOI:** 10.1002/ccr3.7139

**Published:** 2023-03-31

**Authors:** Kento Shionoya, Makoto Kako

**Affiliations:** ^1^ Shonan Gastroenterology Medicine Center Shonan Kamakura General Hospital Kamakura Kanagawa Japan

**Keywords:** arteriovenous malformation, atrial septal defect, heart failure

## Abstract

Hepatic arteriovenous malformation occurs hyperpulsatile heart failure, hepatic encephalopathy, and ascites as clinical symptoms. Oral medication are effective, but hepatic angioembolization or heart surgery in case of its symptoms worsen.

## Graphical abstract

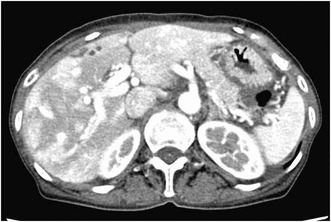

## 1 CASE

Hepatic arteriovenous malformation mostly occurs as a manifestation of hereditary hemorrhagic telangiectasia. Clinical symptoms include hyperpulsatile heart failure, hepatic encephalopathy, and ascites. We present a case of hepatic arteriovenous malformation and atrial septal defect causing heart failure.

**FIGURE 1:**
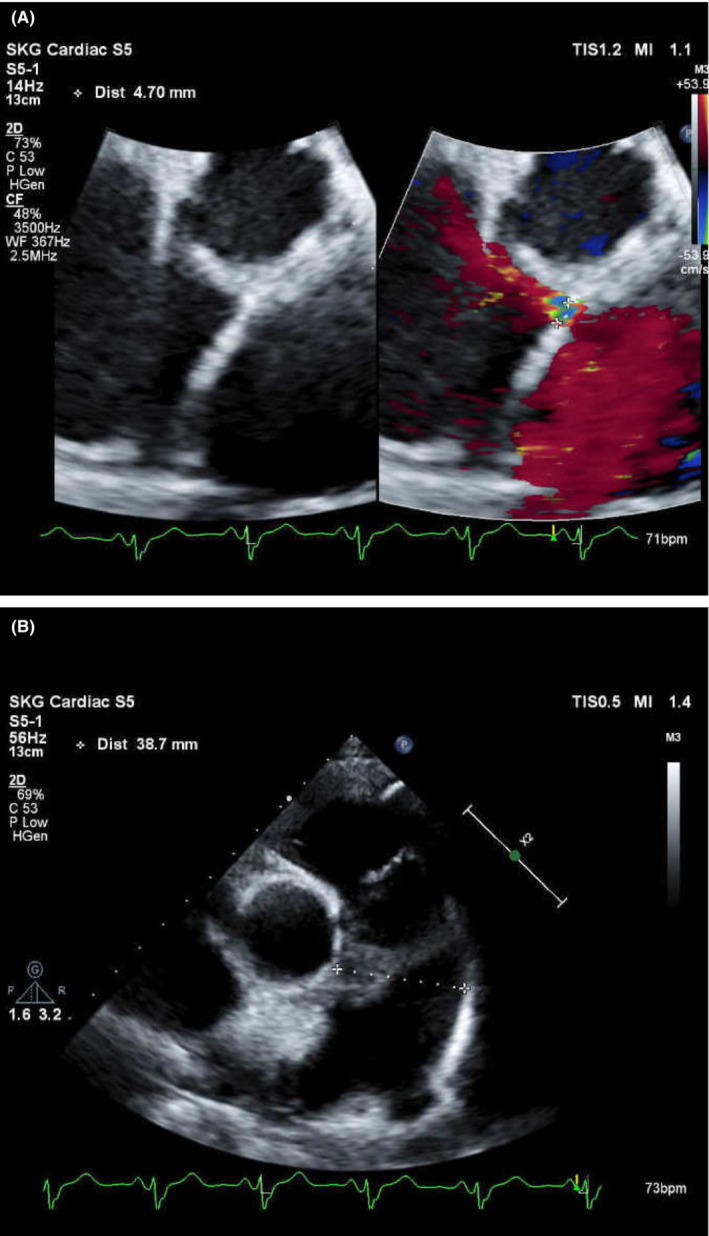
(A, B) Echocardiogram revealed atrial septal defect and pulmonary artery dilation.

**FIGURE 2:**
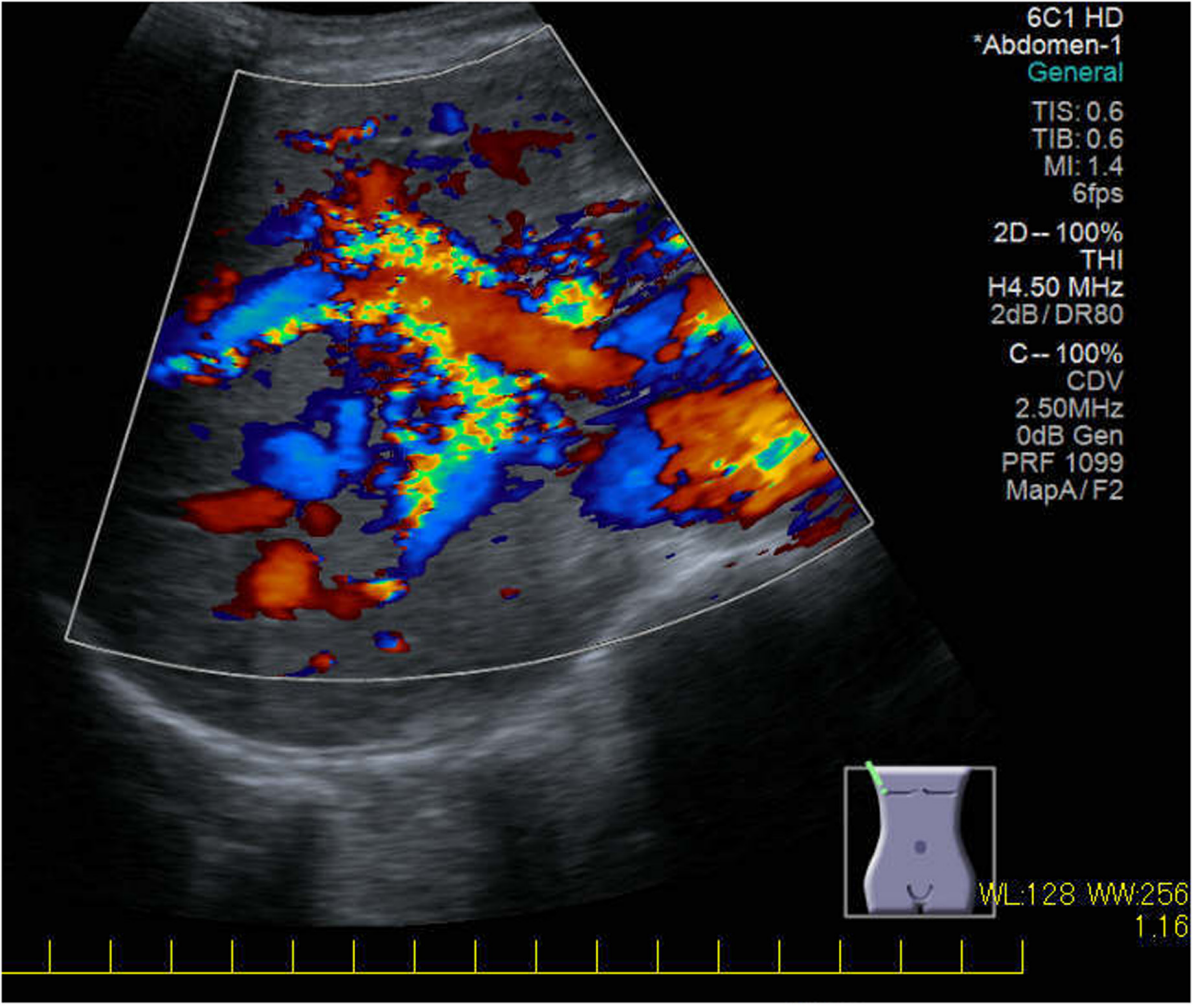
Abdominal ultrasonography revealed that the intrahepatic portal vein was prominent and increased blood flow.

**FIGURE 3:**
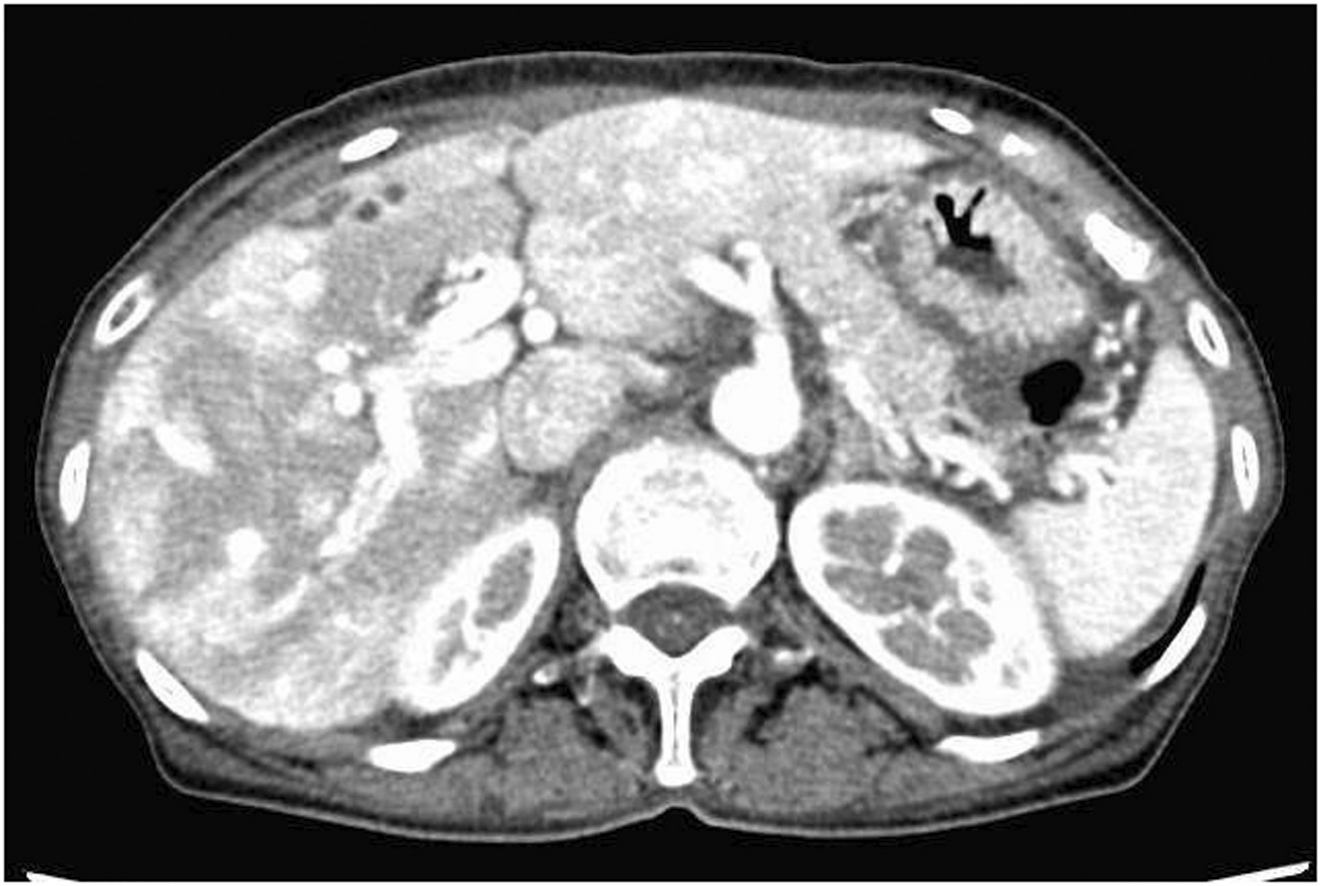
Contrast‐enhanced CT showed multiple hepatic arteriovenous malformations.

A 77‐year‐old woman visited our hospital because of bilateral leg edema and mild dyspnea. Blood test results showed normal liver function and albumin levels, but high levels of ammonia. An echocardiogram revealed atrial septal defect (ASD) and a right–left shunt associated with ASD caused pulmonary artery dilation (Figure 1A,B). Cardiac catheterization revealed pulmonary artery pressure was within the normal range. Abdominal ultrasonography revealed that the intrahepatic portal vein was prominent and increased blood flow, but no findings were suggestive of liver cirrhosis (Figure 2). A contrast‐enhanced CT showed multiple hepatic arteriovenous malformations (AVM) (Figure 3). It was thought that AVM and ASD caused capacitive loading of right heart system and heart failure symptoms had occurred. The cause of high ammonia level was thought to be hepatic AVM. Her leg edema and dyspnea improved, and ammonia levels decreased with oral lactulose and furosemide. Increased cardiac workload may exacerbate symptoms and require surgical intervention in the future, so careful observation is necessary.

Hepatic AVM is a rare disease, mostly occurring as a manifestation of hereditary hemorrhagic telangiectasia. Clinical symptoms include hyperpulsatile heart failure, hepatic encephalopathy, and ascites.^1^, ^2^ Hepatic arterial embolization has been reported to be effective and to improve heart failure symptoms.^3^ Hepatic angioembolization or heart surgery may be necessary in this case if heart failure symptoms worsen.

## AUTHOR CONTRIBUTIONS

**Kento Shionoya:** Conceptualization; data curation; investigation; writing – original draft; writing – review and editing. **Makoto Kako:** Supervision.

## FUNDING INFORMATION

None.

## CONFLICT OF INTEREST STATEMENT

The authors have no conflicts of interest to declare.

## CONSENT

Written informed consent was obtained to publish this report in accordance with the journal's consent policy.

## Data Availability

The technical appendix, statistical code, and dataset are available from the corresponding author upon request. No additional data are available.

## References

[1] Gong B , Baken LA , Julian TM , Kubo SH . High out‐put heart failure due to hepatic arteriovenous fistula during pregnancy. Obstet Gynecol. 1988;72:440‐442. 3405561

[2] Brohee D , Franken P , Fievez M , et al. Highout‐put right ventricular failure secondary to hepatic arteriovenous micro fistulae. Arch Intern Med. 1984;144:1282‐1284. 6732386 10.1001/archinte.1984.00350180230033

[3] Rosch J , Petersen BD , Hall LD , et al. Interventional treatment of hepatic arterial and venous pathology: a commentary. Cardiovasc Intervent Radiol. 1990;13:183‐188. 2121346 10.1007/BF02575471

